# m^6^A modification mediates SLC3A2/SLC7A5 translation in 3-methylcholanthrene-induced uroepithelial transformation

**DOI:** 10.1007/s10565-024-09846-9

**Published:** 2024-01-25

**Authors:** Bixia Liu, Yifan Lv, Wenyu Hu, Yapeng Huang, Xiaoling Ying, Cong Chen, Haiqing Zhang, Weidong Ji

**Affiliations:** 1 Center for Translational Medicine, The First Affiliated Hospital, Sun Yat-sen University, Guangzhou, 510080 China; 2 Department of Pathology, The First Affiliated Hospital, Sun Yat-sen University, Guangzhou, Guangdong, 510080, China; 3 Department of Urology, The First Affiliated Hospital of Guangzhou Medical University, Guangzhou, 510080 China; 4 Department of Urology, The Second Affiliated Hospital of Guangzhou Medical University, Guangzhou, 510080 China

**Keywords:** 3-Methylcholanthracene, Carcinogenesis, SLC3A2, SLC7A5, m^6^A

## Abstract

3-Methylcholanthracene (3-MC) is one of the most carcinogenic polycyclic aromatic hydrocarbons (PAHs). Long-term exposure to PAHs has been thought of as an important factor in urothelial tumorigenesis. N^6^-methyladenosine (m^6^A) exists widely in eukaryotic organisms and regulates the expression level of specific genes by regulating mRNA stability, translation efficiency, and nuclear export efficiency. Currently, the potential molecular mechanisms that regulate m^6^A modification for 3-MC carcinogenesis remain unclear. Here, we profiled mRNA, m^6^A, translation and protein level using “-omics” methodologies, including transcriptomes, m^6^A profile, translatomes, and proteomics in 3-MC-transformed urothelial cells and control cells. The key molecules SLC3A2/SLC7A5 were screened and identified in 3-MC-induced uroepithelial transformation. Moreover, SLC7A5/SLC3A2 promoted uroepithelial cells malignant phenotype in vitro and in vivo. Mechanically, METTL3 and ALKBH5 mediated m^6^A modification of SLC3A2/SLC7A5 mRNA in 3-MC-induced uroepithelial transformation by upregulating the translation of SLC3A2/SLC7A5. Furthermore, programmable m^6^A modification of SLC3A2/SLC7A5 mRNA affected the expression of its proteins. Taken together, our results revealed that the m^6^A modification-mediated SLC3A2/SLC7A5 translation promoted 3-MC-induced uroepithelial transformation, suggesting that targeting m^6^A modification of SLC3A2/SLC7A5 may be a potential therapeutic strategy for bladder cancer related to PAHs.

**Supplementary Information:**

The online version contains supplementary material available at 10.1007/s10565-024-09846-9.

## Introduction

Carcinogens are substances originating from natural and man-made environments that, under certain conditions, can cause cancer in people and animals. The mechanism by which chemical carcinogens induce human tumors is very complicated. Minor carcinogens can induce tumors directly after entering the body, but the majority require metabolic activation or biotransformation before they become carcinogens and have carcinogenic activity (Fishbein et al. 2021; Hecht 2003). Carcinogens cause alterations at the gene, RNA, and protein levels, resulting in the loss of normal growth-regulating activities and the malignant transformation of cells (Smith et al. 2016). The most common and diverse environmental carcinogens are polycyclic aromatic hydrocarbons (PAHs) (Barnes et al. 2018). They are closely related to the health of human beings and can induce cancers in many organs such as the skin (Baudouin et al. 2002), lung (Moorthy et al. 2015), and bladder (Rota et al. 2014) under certain conditions. PAHs are indirect carcinogens in that they are metabolized to produce carcinogens, such as dihydroethylene glycol epoxyphenyl pyrene, when they enter the body. The carcinogenic metabolites then covalently bind to DNA, causing DNA damage, inducing genetic mutations, and ultimately leading to tumor formation. The most widely studied PAHs include benzo[a]pyrene (BaP) and 9,10-dimethylbenzanthracene (DMBA), followed by the highly carcinogenic 3-methylcholanthrene (3-MC). 3-MC could induce tumors in a range of species in vivo and is frequently used in a variety of animal models of induced cancers, including mice models of fibrosarcoma (Cohen et al. 2010), lung cancer (Brown et al. 1999; Wang et al. 2016), and skin cancer (Slaga et al. 1979). Furthermore, 3-MC can induce malignant transformation in cultured cells and is frequently used to build malignant cell transformation models such as the human uroepithelial cell transformation model (MC-SV-HUC T2), the hepatocyte transformation model (BNL 1MEA.7R.1), and the human bronchial epithelial (HBE) cell malignant transformation model (Chen et al. 2021; Reznikoff et al. 1988; Kim et al. 2017).

Bladder cancer, one of the most common malignancies worldwide, is the most prevalent malignant disease of urological tumors in China (Siegel et al. 2021). And one of the major risk factors for bladder cancer is occupational exposure to PAHs (Cumberbatch et al. 2018). Studies on the mechanisms of bladder cancer development and 3-MC carcinogenesis have focused on the inactivation of oncogenes, aberrant activation of tumor signaling, and epigenetic modifications of DNA, while studies focusing on RNA epigenetic enzymes and the modifications they produce are still less reported (Dudziec et al. 2012; Hurst et al. 2012). N^6^-methyladenosine (m^6^A) is the most prevalent post-transcriptional modification in eukaryotic cells (He et al. 2019; Huang et al. 2021). m^6^A is widespread in eukaryotes and is involved in the regulation of activities including mRNA stability, mRNA translation efficiency, and mRNA exit efficiency to control the expression of target genes, thus serving as a key component in normal growth and development. In addition, m^6^A also plays a significant role in tumorigenesis, progression, metastasis, drug resistance, and recurrence (He et al. 2019; Wang et al. 2020b). It has been demonstrated that in bladder cancer, especially in patients with poor prognosis, the overall abundance of m^6^A in tumor tissues is significantly increased and the expression levels of m^6^A-related regulators METTL3 and ALKBH5 are significantly abnormal (Han et al. 2019; Yu et al. 2021). METTL3 can affect tumor development by regulating the level of m^6^A modification in mRNA of several key oncogenes or tumor suppressor genes (Zeng et al. 2020). Previous studies on the oncogenic mechanism have demonstrated that the targets of m^6^A are extensive and the mechanisms are complex. In addition, the function and mechanism of m^6^A modifications in 3-MC-induced bladder cancer are not yet clear.

Our study aimed to investigate the role of m^6^A modification in 3-MC-induced malignant transformation of uroepithelial cells. The results of multi-omics analysis, including transcriptome, m^6^A modification profile, and proteomics, revealed that SLC3A2/SLC7A5 are key target genes during 3-MC-induced uroepithelial transformation. Mechanically, METTL3 and ALKBH5-mediated m^6^A modification plays an important role in 3-MC-induced uroepithelial transformation by upregulating the translation of oncogenes SLC3A2 and SLC7A5, thus revealing a novel mechanism for bladder carcinogenesis at the molecular level. Our data suggests that targeting METTL3/ALKBH5 and their downstream genes SLC3A2/SLC7A5 may be a potential therapeutic strategy for bladder cancer.

## Materials and methods

### Plasmid construction

The Human Umbilical Vein Endothelial Cells (HUVEC) genome served as a PCR amplification template to obtain the target fragment to be ligated (primer sequences see Table S1). The empty overexpression plasmid LentiORF PLEX-MCS vector and purified PCR amplified fragments were digested by restriction enzymes NotI and BamHI and subsequently ligated by T4 ligase to obtain the SLC3A2 or SLC7A5 over-expression plasmid. Besides, SLC3A2 or SLC7A5 knockout plasmid were constructed using lentiCRISPR v2 which was obtained from Feng Zhang (Addgeneplasmid#52961). The gRNAs of SLC3A2 or SLC7A5 were designed by the CRISPR online design tool (http://chopchop. cbu.uib.no/index.php). In this study, 3 pairs of gRNAs with minimum off-target effects and the highest scores were selected (listed in Table S2).

The seamless cloning primer design tool (http://123.56.75.195/) was used to design the wild-type and mutant-type fragments according to METLL3 catalytic domain (METTL3CD) and ALKBH5 catalytic domain (ALKBH5CD), and then the fragments were respectively synthesized and amplified by PCR. The EF1a-dCasRx-2A-EGFP vector was digested with NheI endonuclide and then seamlessly connected with the PCR amplified fragment to obtain EF1a-dCasRx-2A-WT-METTL3 activity region-EGFP, EF1a-dCasRx-2A-mut-METTL3 activity region-EGFP, EF1a-dCasRx-2A-WT-ALKBH5 activity region-EGFP, and EF1a-dCasRx-2A-mut-ALKBH5 activity region-EGFP. The gRNAs targeting SLC3A2 or SLC7A5 were designed according to m^6^A modification site on mRNA (see Table S3). The gRNA oligos were phosphorylated and annealed. Lentiguide-Hygro-mTagBFP2 vector was digested with BSMBI and PspXI endonucrenases and ligated with sgRNA scaffold-cppt/cts fragment by T4 DNA ligase. The ligated productions were digested again with BsmBI and then ligated with the treated gRNA oligos to construct a guide RNA (gRNA) expression vector which target SLC3A2 or SLC7A5 mRNA 3'UTR region.

### Cells transfection

Cells were cultured on petri dishes in DMEM complete medium without Penicillin-Streptomycin. When cells grow to 90–95% confluency, the transfection system was prepared according to the instructions of Lipofectamine 3000 (Thermo Fisher Scientific). For each group, cells per dish were transfected with 12 ug of target plasmid and 3 ug of each packaging plasmid pCMV-VSVG, TAT, RAII, HEPM2. In order to establish a stable cell line, the target cells were co-cultured with lentivirus mentioned above and 8 μg/ml polybrene (Sigma-Aldrich) for 48 h. Subsequently, 1 μg/ml puromycin (Sigma-Aldrich) was used to screen the successfully transfected cells, and a Western blot was used for detecting the overexpression or knockout efficiency.

### Methylated RNA immunoprecipitation (MeRIP)

Total RNA of cells was fragmented in Fragmentation Reagent (Ambion) at 94°C for 5 min. And then the fragmented RNA was purified according to the instruction of Oligo Clean & Concentrator kit (ZYMO RESEARCH). About 100 ng fragmented RNA was used to construct the input library, and the rest was used for immunoprecipitation. A total of 25 μl of Pierce™ Protein A/G Magnetic Beads (Thermo Fisher Scientific) and 6 ul of 0.5 μg/μl anti-m^6^A primary antibody (202003, Synaptic Systems) were mixed in 1×IP buffer and incubated for 3 h at 4 °C in advance. And then the mixture was incubated with the fragmented mRNA which was prepared for immunoprecipitation overnight at 4 °C. After 5 times of strict washing, the captured RNA was eluted by competition with N6-methyladenosine (Sigma-Aldrich).

### Polysome profiling

Cells were collected and lysed in polysome lysis buffer (150 mM NaCl, 50 mM MOPS, 15 mM MgCl_2_, 0.5% Triton X-100, 100 μg/ml cycloheximide, 200 U RNaseOUT, 2 mM PMSF, 1 mg/ml heparin, and 1 μM benzamine) on ice for 10 min. The lysis solution was centrifugated at 12,500×g for 10 min at 4 °C, and the supernatant was taken as the prepared sample. And then the samples were carefully added to a 10–50% sucrose gradient along the tube wall and centrifuged at 36,000 rpm for 2.5 h at 4 °C. The mRNAs in the monosomal fraction and the polysomal fraction were obtained and extracted for RT-qPCR.

### Single-base detection of m^6^A sites

A total of 300 ng total RNA was incubated with 20 nM synthesized Probe R and Probe L in the 7-μl reaction mixture of T3 DNA ligation buffer at 85 °C for 3 min and then 35 °C for 10 min (Table S4). 50U T3 DNA ligase (New England Biolabs) with an appropriate amount of ligation buffer was added into the RNA mixtures to the final volume of 10 μl. This mixture was incubated at 35 °C for 10 min, chilled on ice immediately, and then amplified by PCR. The amount of PCR products which could be used to assess the ligation efficiency was measured by agarose gel electrophoresis and RT-qPCR.

### Animal experiments

The procedures of animal care and experiments were approved by the Institutional Ethics Committee for Clinical Research and Animal Trials of the First Affiliated Hospital of Sun Yat-sen University. The 4-week-old BALB/c mice were obtained from the Model Animal Research Center of Nanjing University (MARC). MC-SV-HUC T2 cells were stably transfected with SLC3A2 knockout lentivirus (KO-SLC3A2 cells), SLC7A5 knockout lentivirus (KO-SLC7A5 cells) or control lentivirus (NC). And the three kind of cells were subcutaneously injected into three groups of mice separately. Tumor formation was then monitored every week, and the tumors were removed, weighed, fixed, and embedded for IHC after 4 weeks.

### IHC analysis

Tissues from xenograft tumor samples were fixed in 4% formalin and prepared into 5 μm paraffin-embedded sections. Tissue sections were defatted in xylene and rehydrated by graded alcohol baths. Sections were first subjected to antigen retrieval by simply immersing the sections in EDTA antigen retrieval buffer and microwave treatment, followed by removing endogenous peroxidase activity using 3% methanolic hydrogen peroxide and incubating in 5% bovine serum albumin for blocking nonspecific binding. Subsequently, these tissues were incubated with 1:100 diluted anti-KI67 (#9027, Cell Signaling Technology), anti-SLC7A5 (ab305251, Abcam) antibodies, and anti-SLC3A2 (ab307587, Abcam) overnight at 4 °C, and then incubated with the secondary antibody.

### Statistical analysis

All assays had three duplicates. The data were statistically analyzed via GraphPad Prism 9.0 and SPSS 16.0 (IBM). ANOVA or the *t*-test was used for analyzing the relationship between multiple sets of continuous variables. All data was shown as mean ± SEM, with *P*<0.05 deemed statistically significant.

## Results

### Multi-omics analysis in MC-induced urothelial transformation

To investigate the novel transcriptome changes in MC-induced urothelial transformation, we performed transcriptome sequencing (RNA-Seq) in SV-HUC-1 and MC SV-HUC T2 cells. A total of 4762 differentially expressed genes (DEGs) with False Discovery Rate (FDR) no more than 0.05 and |log_2_ Fold Change| greater than 1 were screened out. Compared to the SV-HUC-1 group, MC SV-HUC T2 had 2428 genes upregulated while 2334 DEGs significantly downregulated (Fig. S1A). To understand the biological function of these DEGs, we conducted GO and KEGG pathway analysis. BP analysis showed that the DEGs were enriched in extracellular organization, CC analysis showed the DEGs were abundant in collagen-containing extracellular matrix and cell−cell junction, while MF analysis indicated the DEGs were mainly function in extracellular matrix structural constituent and integrin binding (Fig. S1B). Moreover, KEGG analysis suggested that the DEGs were linked to some cancer-related pathways, such as PI3K−Akt signaling pathway and MAPK signaling pathway (Fig. S1C), suggesting MC-induced urothelial transformation leading to core carcinogenic processes altered in the human uroepithelial cells.

To explore the profile by which MC-induced urothelial transformation regulates m^6^A modification, an m^6^A-modified RNA immunoprecipitation sequence (MeRIP-seq) was conducted in the cells. TopHat was used to map reads to the genome reference sequence. Compared with the normal cells, there were 730 m^6^A peaks on the transcripts of 659 genes that were significantly upregulated, and 246 m^6^A peaks on the transcripts of 232 genes were downregulated in the malignant transformation cells (Fig. S1D). Interestingly, these altered m^6^A genes were mainly enriched in protein folding, protein processing, and carcinogenic pathway according to the analysis results of GO and KEGG terms (Fig. S1E–F).

Translation is a critical step in gene expression regulation and also one of the energy-intensive biological activities in the cell. Translation dysregulation induced by abnormal function of upstream signaling pathways and/or alterations in the translation machinery components is frequent in the malignant transformation process (Bjornsti and Houghton 2004; Sun et al. 2021). Therefore, we depicted the translation and protein profiles by Ribo-seq analysis and proteome analysis. Using ribosome profiling sequencing, we detected a total of 5435 genes with changed translation levels in the MC SV-HUC T2 group (Fig. S2A). GO analysis of the altered genes revealed a strong enrichment for signal transduction and transmembrane transport (Fig. S2B). Further, KEGG analysis showed differential gene enrichment pathways involving Axon guidance, ECM-receptor interaction, focal adhesions, Hippo signaling pathway, and Rap1 signaling pathway (Fig. S2C).

A total of 699 proteins were differentially expressed between transformed cells and control cells. Of these, 331 proteins were upregulated and 368 were downregulated (Fig. S2D). The differentially expressed proteins (DEPs) were grouped into various biological processes, with the majority comprising neutrophil activation involved in the immune response, the ribose phosphate metabolic process, the ATP metabolic process, and cell redox homeostasis. The MF of these proteins is important in cadherin binding and unfolded protein binding. The CC analysis showed that these proteins were mainly in phagocytic vesicles, melanosomes, focal adhesions, secretory granule lumen, and mitochondrial matrix (Fig. S2E). KEGG pathway analysis showed Salmonella infection, protein processing in the endoplasmic reticulum (ER), pathogenic Escherichia coli infection, and tight junction were significantly involved (Fig. S2F).

We have described the hallmark of 3-MC malignant transformation at multiple levels including transcription, m^6^A, translation and protein levels. While single-level OMICS techniques have assisted to identify epigenetic alterations and molecular subtyping of tumors based on gene/protein expression, they lack the capacity to prove the causal link between molecular signatures and the phenotypic manifestation of cancer hallmarks. Multi-omics techniques, on the other hand, offer the potential to unveil the complicated molecular process underpinning diverse phenotypic manifestations of cancer hallmarks such as tumorigenesis and metastasis. The analysis integrating mRNA and translation levels, which aimed to explore the translation efficiency (TE), showed that the TE of 6120 genes was significantly changed (Fig. S3A). The genes were entered into the DAVID 6.8 database for GO enrichment analysis and KEGG pathway enrichment analysis. We observed the enrichment of terms linked to signaling, biological regulation, and metabolic process (Fig. S3B–C). It is suggested that the induction of 3-MC may exert carcinogenesis through the above pathways. m^6^A methylation modification was proven to alter the progression of tumor cells via weakening mRNA stability, increasing TE and nucleation efficiency (Deng et al. 2018; Jin et al. 2019; Lin et al. 2016; Yang et al. 2019). To identify what roles did m^6^A play in the process of 3-MC-induced uroepithelial transformation, transcriptome or translatome datasets were integrated with m^6^A datasets, respectively, to analysis. After 3-MC treatment, there were 190 genes with co-changes at mRNA and m^6^A levels and 136 genes with co-changes at TE and m^6^A levels (Fig. S4A–B). GO enrichment analysis implicated that these genes were primarily enriched in positive regulation of transcription by RNA polymerase II and cadherin binding (Fig. S4C). KEGG analysis also showed tight junction and protein processing in the endoplasmic reticulum (Fig. S4D). Taken together, it was suggested that m^6^A-regulated 3-MC-induced uroepithelial transformation may be dependent in part on its abilities to affect stability and TE of mRNA.

**Fig. 1:**
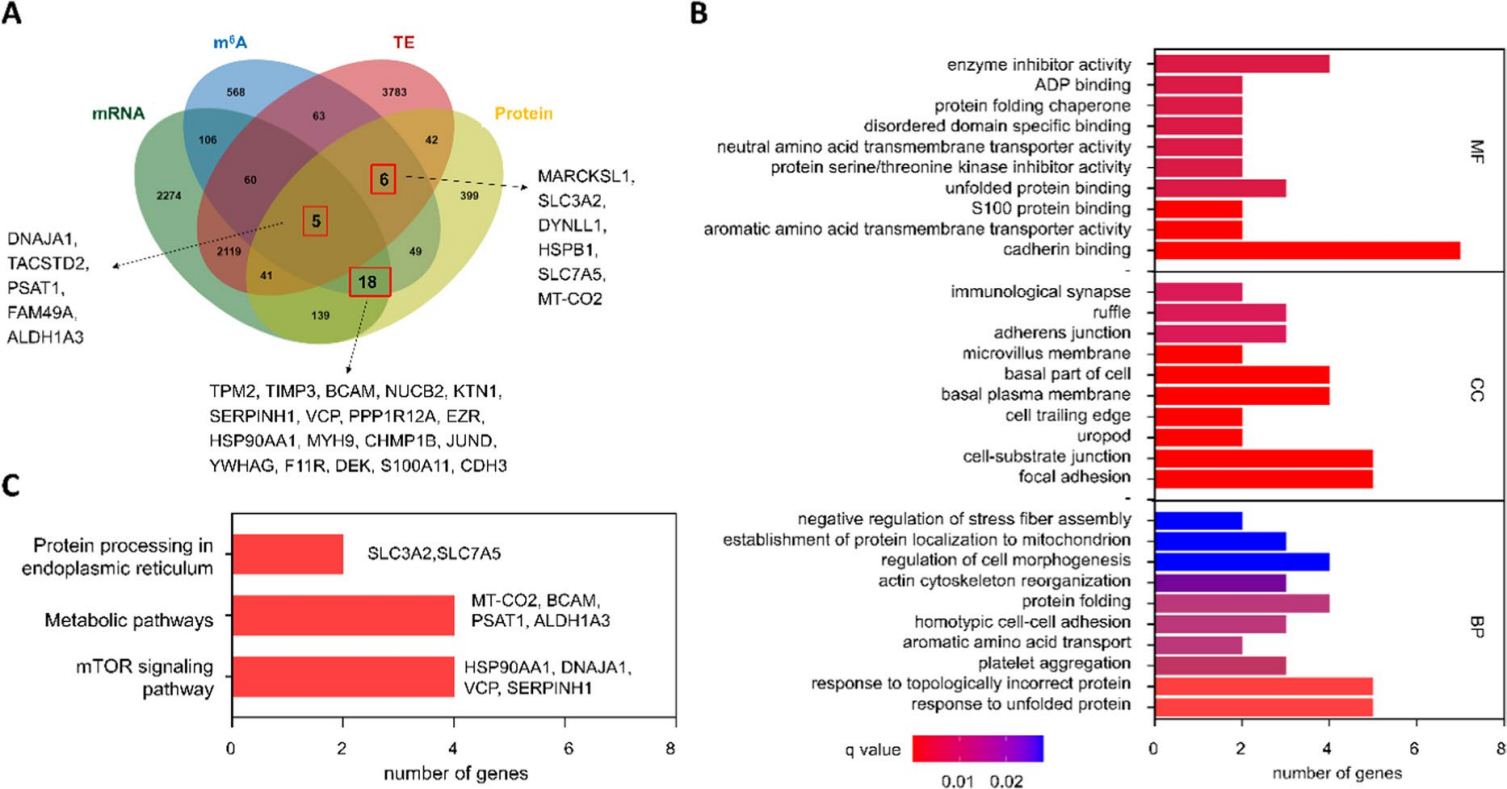
Integrated analysis of muti-omics. **A** Venn diagram displaying overlapping significantly regulated genes at mRNA, m^6^A, translation, and protein levels (https://bioinfogp.cnb.csic.es/tools/venny/index.html). **B** GO function analysis of the key genes. **C** KEGG pathway enrichment analysis based on the key genes

Next, we focused on the function of m^6^A in regulating stability and TE of mRNA and aimed to seek their key downstream gene. We brought the protein sequencing data of these genes into the integrated analysis, given that most genes perform their functions by directing the synthesis of proteins. Based on the integrated analysis, 29 genes were selected according to the conditions: (1) mRNA levels or translation efficiency were significantly changed; (2) protein levels were significantly changed; (3) m^6^A levels were significantly changed (Fig. 1A, Table 1). Fifteen genes were removed from the list of candidate genes due to their abnormal protein expression caused by post-translational modification or other m^6^A-independent factors. Following 3-MC therapy, the mRNA expression and m^6^A modification of certain genes involved in protein processing in the endoplasmic reticulum, including HSP90AA1, DNAJA1, and VCP, were changed. Furthermore, the expressions of MT-CO2, BCAM, PSAT1, and ALDH1A3 were substantially different between the two groups, indicating that both vitamin B6 metabolism and amino acid anabolism/catabolism were dysregulated in SV-HUC-1 cells following 3-MC therapy (Fig. 1B, C).

**Table 1:** MeRIP-Seq and iTRAQ mass spectrometry data of the 14 candidate genes

Gene	m^6^A-|log_2_FC|	Protein-|log_2_FC|
FAM49A	1.11	0.673
SLC3A2	**1.04**	**0.529**
BCAM	0.924	0.345
PSAT1	0.804	0.264
MT-CO2	0.529	0.299
KTN1	0.528	0.353
NUCB2	0.497	0.285
CDH3	0.445	0.307
F11R	0.423	0.393
PPP1R12A	0.393	0.264
TACSTD2	0.312	0.599
YWHAG	0.221	0.266
SLC7A5	**0.205**	**0.349**
ALDH1A3	0.148	1.031

### Identification of key molecules SLC3A2/SLC7A5 in 3-MC-induced uroepithelial transformation

**Fig. 2:**
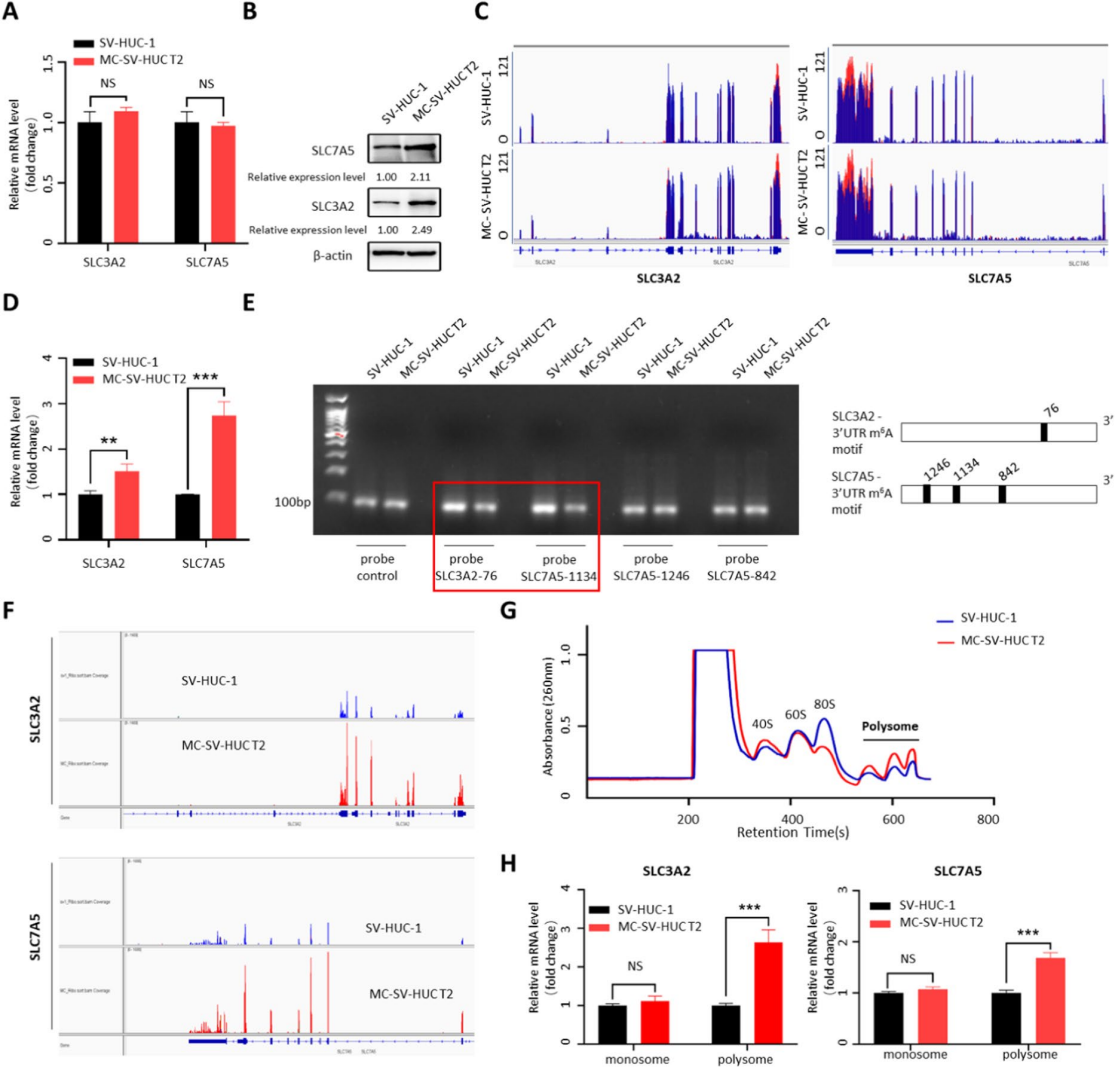
Validation of multi-omics sequencing results for SLC3A2 and SLC7A5. **A** RT-qPCR analysis of SLC3A2 and SLC7A5 mRNA in SV-HUC-1 cells and MC-SV-HUC T2 cells. **B** Western blotting of SLC3A2 and SLC7A5 expression in SV-HUC-1 cells and MC-SV-HUC T2 cells. Relative expression level =$\frac{\ \textrm{group}\ \left(\textrm{target}\ \textrm{protein}\textrm{s}/\textrm{control}\ \textrm{protein}\right)}{\textrm{control}\ \textrm{group}\ \left(\textrm{target}\ \textrm{protein}\textrm{s}/\textrm{control}\ \textrm{protein}\right)}$ **C** m^6^A sites in the SLC3A2 and SLC7A5 mRNA 3′UTR are shown using the MeRIP-Seq data in SV-HUC-1 and MC-SV-HUC T2 cells. **D** m^6^A enrichment in the SLC3A2 and SLC7A5 mRNA 3′UTR was validated by MeRIP-qPCR in SV-HUC-1 and MC-SV-HUC T2 cells. **E** m^6^A enrichment in the SLC3A2 and SLC7A5 mRNA 3′UTR was detected by single-base mapping of m^6^A. **F** The translation levels of SLC3A2 and SLC7A5 were shown using the Ribo-Seq data. **G** Polysome profile of SV-HUC-1 and MC-SV-HUC T2 cells. **H** RT-qPCR analysis of SLC3A2 and SLC7A5 mRNA derived from Polysome profiling. (**P<0.01, ***P<0.001)

According to the results of the multi-omics comprehensive analysis, the changes of SLC3A2 and SLC7A5 were most obvious during the 3-MC-induced uroepithelial transformation (Table 1). Interestingly, we found that the proteins encoded by SLC7A5 and SLC3A2 are bound to form a heterodimeric plasma membrane protein with extracellular disulfide to jointly play the role of amino acid transport. So SLC3A2 and SLC7A5 were selected as candidate targets for further investigation. To explore the biological function of 3-MC on SLC3A2/SLC7A5, we analyzed the SLC3A2/SLC7A5 mRNA, translation efficiency, protein, and m^6^A levels in MC-SV-HUC T2 and SV-HUC-1 cells. Compared to the control group, the mRNA levels of SLC3A2/SLC7A5 were not significantly upregulated after 3-MC treatment (Fig. 2A), while the protein levels significantly increased (Fig. 2B). Meanwhile, MeRIP-seq results showed the presence of m^6^A modifications in these two genes, and the m^6^A levels were also significantly enriched in after 3-MC treatment (Fig. 2C–E), which is consistent with previous studies showing that m^6^A methylation modifications affect translation efficiency. This raises the possibility that m^6^A methylation modifications may increase SLC7A5 and SLC3A2 protein expression in MC-SV-HUC T2 cells in a post-transcriptional manner.

To explore the specific m^6^A sites mentioned above, we used T3 DNA ligase, which has a significant steric hindrance effect on m^6^A modification sites. According to the previous m^6^A single nucleotide resolution m^6^A iCLIP (miCLIP)-Seq data, we retrievalled one SLC3A2 and three SLC7A5 m^6^A sites, with the unmodified A site serving as a negative control. As can be seen from Fig. 2E, the ligation efficiency of T3 ligase on SLC3A2-76 site and SLC7A5-1134 site was significantly inhibited after 3-MC treatment, indicating that 3-MC was able to increase the m^6^A modification on SLC3A2-76 site and SLC7A5-1134 site. Subsequently, our Ribo-seq sequencing data showed that the translation efficiency of SLC3A2 and SLC7A5 were significantly higher in MC-SV-HUC T2 cells than in control cells (Fig. 2F). In addition, polysome profile assay revealed that the overall translation levels of MC-SV-HUC T2 were increased (Fig. 2G). The mRNAs in the monosomal fraction combine one ribosome and have very low translation efficiency, so they were used as the reference to correct the initial total mRNA amount (Stark et al. 2019). SLC3A2 and SLC7A5 mRNA detected at a significantly increased abundance in the polysomal fraction showed that the translation efficiency of SLC3A2 and SLC7A5 was significantly up-regulated after 3-MC treatment (Fig. 2H), in agreement with the ribosome profiling results. Taken together, these results suggest that 3-MC treatment of human uroepithelial cells can increase the m^6^A modification on SLC3A2-76 site and SLC7A5-1134 site, improve the translation efficiency, and increase the protein expression level.

### SLC7A5 and SLC3A2 promote uroepithelial cells transformation and tumorigenesis

**Fig. 3:**
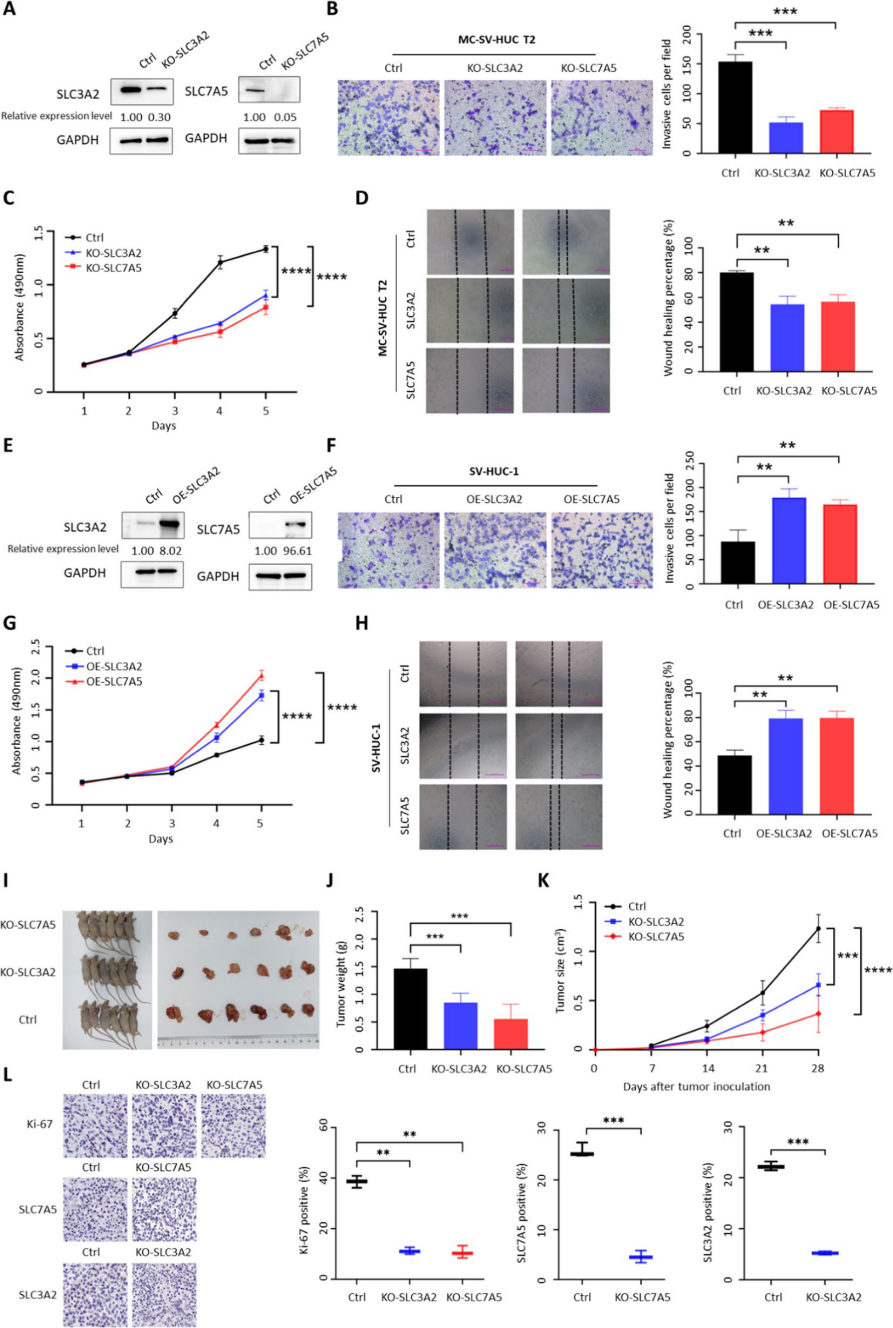
SLC3A2 and SLC7A5 promoted the growth and progression of bladder cancer cells in vitro and in vivo. **A** SLC3A2 and SLC7A5 were knocked out in MC-SV-HUC T2 cells. **B** Transwell assay to detect the effects of knocked out of SLC3A2 and SLC7A5 on invasion ability of MC-SV-HUC T2 cells. **C** MTS assay to detect the effects of knocked out of SLC3A2 and SLC7A5 on proliferation ability of MC-SV-HUC T2 cells**. D** Scratch assay to detect the effects of knocked out of SLC3A2 and SLC7A5 on migration ability of MC-SV-HUC T2 cells**. E** SLC3A2 and SLC7A5 were overexpressed in SV-HUC-1 cells. **F** Transwell assay to detect the effects of over-expression of SLC3A2 and SLC7A5 on invasion ability of SV-HUC-1 cells. **G** MTS assay to detect the effects of over-expression of SLC3A2 and SLC7A5 on proliferation ability of SV-HUC-1 cells. **H** Scratch assay to detect the effects of over-expression of SLC3A2 and SLC7A5 on migration ability of SV-HUC-1 cells. **I** Representation picture of tumor formation of xenograft in nude mice. **J** Weights of tumors in three groups were measured at the end point. **K** Summary of tumor volume of mice, which were measured every 7 days. **L** Representative images of SLC3A2, SLC7A5, and Ki67 IHC staining in tumors. Data are presented as ± SEM; *n* = 3. **p < 0.01, ***p < 0.001, ****p < 0.0001

Previous studies indicated that SLC3A2 and SLC7A5 exert functions through activating mTOR pathway in human tumors, thereby promoting growth and metastasis. To explore the functional role of SLC3A2 and SLC7A5 in the malignant transformation of 3-MC, we established stable overexpression or knockout models in normal and malignantly transformed cells. As expected, knockout of SLC3A2 and SLC7A5 remarkably suppressed the proliferation, migration, and invasion ability of MC SV-HUC T2 cells (Fig. 3A–D). In addition, overexpression of SLC3A2 and SLC7A5 greatly accelerated the proliferation, migration, and invasion of SV-HUC-1 cells (Fig. 3E–H). To further validate the oncogenic function of SLC3A2 and SLC7A5 in MC SV-HUC T2, we performed a subcutaneous implantation assay in nude mice to verify the effect of SLC3A2 and SLC7A5 knockout on tumorigenicity. We observed that stable knockout of SLC3A2 and SLC7A5 effectively inhibited tumor growth in vivo, as reflected by a significant reduction in tumor size and growth rate compared to non-target CRISPR-V2 controls (Fig. 3I–L). Moreover, Ki67 staining showed that the proliferation ability of tumor cells in vivo was also significantly inhibited after knockout of SLC3A2 and SLC7A5 (Fig. 3L). Therefore, our results indicate that SLC3A2/SLC7A5 exert the enormous function on promoting the malignant progression of bladder cancer cells in vitro and vivo.

### m^6^A modification regulated SLC3A2/SLC7A5 translation in 3-MC-induced uroepithelial transformation

**Fig. 4.**
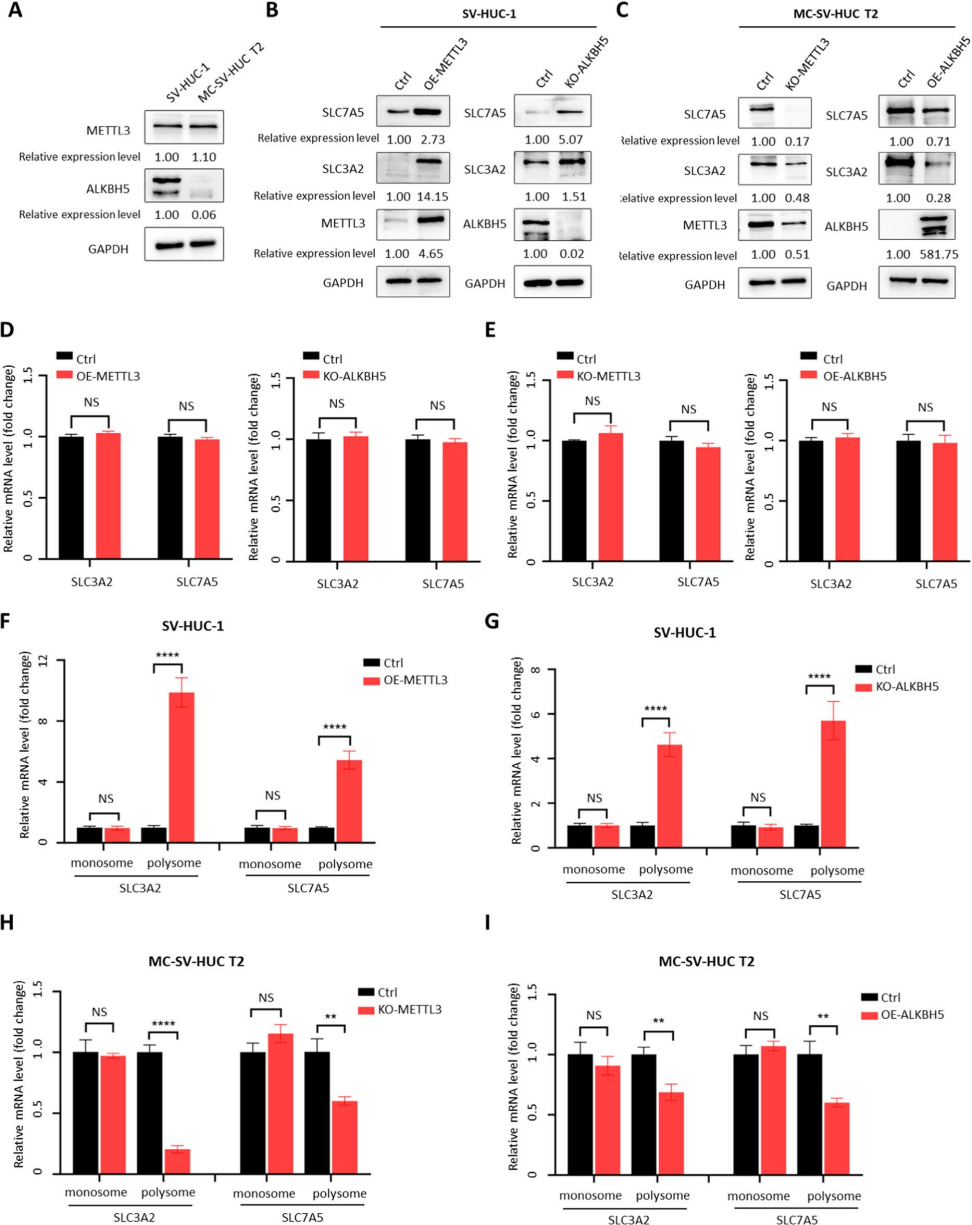
METTL3 and ALKBH5 regulate m^6^A modification within the 3′ UTR of SLC3A2/SLC7A5 and SLC3A2/SLC7A5 translation. **A** Western blotting of METTL3 and ALKBH5 expression in SV-HUC-1 cells and MC-SV-HUC T2 cells. **B** Western blotting to verify the effect of METTL3 overexpression or ALKBH5 knockout on SLC3A2 and SLC7A5 expression in SV-HUC-1 cells. **C** Western blotting to verify the effect of METTL3 knockout or ALKBH5 overexpression on SLC3A2 and SLC7A5 expression in MC-SV-HUC T2 cells. **D** RT-qPCR results of SLC3A2 and SLC7A5 in METTL3-overexpressed or ALKBH5-knocked out SV-HUC-1 cells. **E** RT-qPCR results of SLC3A2 and SLC7A5 in METTL3-knocked out or ALKBH5-overexpressed MC-SV-HUC T2 cells. **F** RT-qPCR analysis of polysome-bound SLC3A2 or SLC7A5 mRNAs in METTL3-overexpressed SV-HUC-1 cells. **G** RT-qPCR analysis of polysome-bound SLC3A2 or SLC7A5 mRNAs in ALKBH5-knocked out SV-HUC-1 cells. **H** RT-qPCR analysis of polysome-bound SLC3A2 or SLC7A5 mRNAs in METTL3-knocked out MC-SV-HUC T2 cells. **I** RT-qPCR analysis of polysome-bound SLC3A2 or SLC7A5 mRNAs in ALKBH5-overexpressed MC-SV-HUC T2 cells

Our previous data revealed that SLC3A2 and SLC7A5 mRNA levels were unchanged, and the translation efficiency, protein levels, and m^6^A modification levels were significantly increased after 3-MC treatment. Previous studies have found that YTHDF1 can selectively recognize m^6^A modifications on specific gene mRNAs to increase their translation efficiency and promote protein expression without affecting their mRNA abundance (Pi et al. 2021). Therefore, we hypothesized that an increase in the level of m^6^A modification leads to enhanced translation efficiency and upregulation of protein expression in SLC3A2 and SLC7A5 mRNAs. To verify this hypothesis, we first measured the expression levels of METTL3 and ALKBH5 in SV-HUC-1 and MC-SV-HUC T2 cells, which act as m^6^A writers and erasers, respectively, using a Western blot assay. Compared to SV-HUC-1 cells, METTL3 expression was slightly increased, while ALKBH5 expression was significantly decreased in MC-SV-HUC T2 cells (Fig. 4A), which is consistent with changes in other types of tumors (Wang et al. 2020a; Zhang et al. 2017). In SV-HUC-1 cells, ALKBH5 knockout or METTL3 overexpression both facilitated the expression of SLC3A2 and SLC7A5 (Fig. 4B). In contrast, in MC-SV-HUC T2 cells, knockout of METTL3 or overexpression of ALKBH5 reduced SLC3A2 and SLC7A5 protein levels (Fig. 4C). However, the mRNA levels of SLC3A2 and SLC7A5 were unchanged regardless of METTL3 and ALKBH5 knockout or over-expression in corresponding cell lines (Fig. 4D, E). Then, translation levels of SLC3A2 or SLC7A5 mRNA in each experimental group were measured using sucrose gradient fractionation. We found that the translation levels of SLC3A2 and SLC7A5 were significantly increased after over-expression of METTL3 or knockout of ALKBH5 (Fig. 4F, G) and decreased after knockout of METTL3 or over-expression of ALKBH5 (Fig. 4H, I). These data show that METTL3 and ALKBH5 modulate the expression levels of SLC3A2 and SLC7A5 via regulating the translation process of mRNA rather than directly affecting their mRNA levels.

### Programmable m^6^A modification of SLC3A2 and SLC7A5 mRNAs promotes its translation

**Fig. 5:**
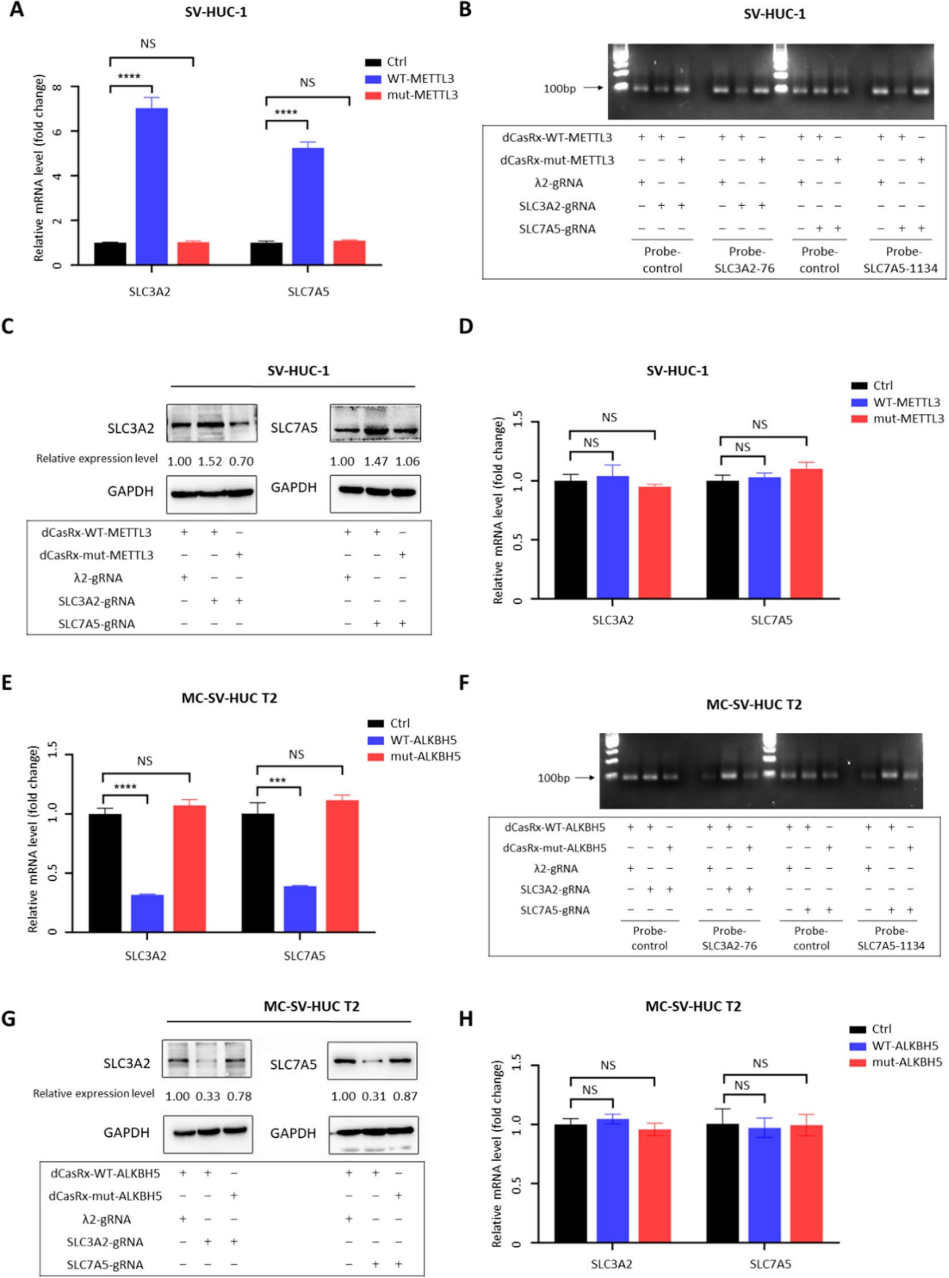
Programmable m^6^A modification of SLC3A2 and SLC7A5 mRNAs promotes its translation. **A** MeRIP showed that the dCasRx-METTL3 fusion protein upregulated the m^6^A levels at SLC3A2-76 site and SLC7A5-1134 site in SV-HUC-1 cells. **B** Single-base detection of m^6^A showed that the dCasRx-METTL3 fusion protein upregulated the m^6^A levels at SLC3A2-76 site and SLC7A5-1134 site in SV-HUC-1 cells. **C** dCasRx-METTL3 fusion protein promoted SLC3A2 and SLC7A5 gene expression in SV-HUC-1 cells. **D** dCasRx-METTL3 fusion protein did not affect the mRNA levels of SLC3A2 and SLC7A5 in SV-HUC-1 cells. **E** MeRIP showed that dCasRx-ALKBH5 fusion protein downregulated the m^6^A levels at SLC3A2-76 site and SLC7A5-1134 site in MC-SV-HUC T2 cells. **F** Single-base mapping of m^6^A showed that dCasRx-ALKBH5 fusion protein downregulated the m^6^A levels at SLC3A2-76 site and SLC7A5-1134 site in MC-SV-HUC T2 cells. **G** dCasRx-ALKBH5 fusion protein inhibited SLC3A2 and SLC7A5 gene expression in MC-SV-HUC T2 cells. **H** dCasRx-ALKBH5 fusion protein did not affect the mRNA levels of SLC3A2 and SLC7A5 in MC-SV-HUC T2 cells

We further investigated the role of these m^6^A sites, including SLC3A2-76 and SLC7A5-1134, in translational regulation. The catalytic structural domains of METTL3 (METTL3CD) and ALKBH5 (ALKBH5CD) were fused separately to catalytically inactive CasRx variants (dCasRx) and designed guide RNAs (gRNAs) for specific sites of target gene mRNAs. Under the guidance of gRNA, the dCasRx fusion protein could bind to the specific sites of target genes, thus achieving bidirectional regulation of m^6^A levels at specific sites in cell. Meanwhile, we generated catalytically inactive METTL3 and ALKBH5 mutants and constructed MUT-METTL3CD-dCasRx fusion protein and MUT-ALKBH5CD-dCasRx fusion protein with unchanged m^6^A levels and expression levels of SLC3A2 and SLC7A5 in cells. m^6^A MeRIP and single-base detection results showed that the dCasRx-METTL3 fusion protein in normal cells could significantly upregulate m^6^A at the SLC3A2-76 site and SLC7A5-1134 site (Fig. 5A, B) and correspondingly promoted gene expression with unchanged mRNA levels (Fig. 5C, D). In addition, the dCasRx-ALKBH5 fusion protein in MC-SV-HUC T2 cells significantly down-regulated the m^6^A levels at the SLC3A2-76 locus and SLC7A5-1134 locus (Fig. 5E, F) and repressed the expression of SLC3A2 and SLC7A5 with the mRNA levels remained unchanged (Fig. 5G, H). Overall, these data suggest that m^6^A modification at SLC3A2-76 site and SLC7A5-1134 site may exert considerable impact on the regulation of the translation process and upregulated protein expression of SLC3A2 and SLC7A5 in the process of 3-MC-induced uroepithelial transformation.

## Discussion

Traditional studies found that the identification of disease biomarkers obtained from single-omics sequencing data resulted in the inability to describe the linkages or interactions between the various histology in the disease and made the exploration of the complex biological mechanisms in the disease extremely limited. The emergence of multi-omics integrated analysis presents an all-encompassing interpretation of the global view of biological processes and the nature of interactions between different histological levels, providing insight into the cancer development and progression (Chu et al. 2021; Hasin et al. 2017; Olivier et al. 2019). In this study, by integrating multi-omics data of the epigenomics, transcriptomics, translomics, and proteomics, we first unveiled the mechanism of the role of m^6^A in the malignant transformation of 3-MC-induced uroepithelial cells.

The SLC3A2 and SLC7A5 genes encode the heavy and light chains of the SLC3A2/SLC7A5 amino acid transport complex, respectively, which are then linked by extracellular disulfide bonds to form heterodimers for amino acid transport functions (Mastroberardino et al. 1998). SLC3A2 and SLC7A5 have been found to be highly expressed in various types of tumors or cancer cells, and they have been shown to exert pro-cancer effects in a variety of cancers through multiple pathways (Furuya et al. 2012; Kaira et al. 2009; Liang et al. 2011). McCracken et al. found that some essential amino acids such as L-leucine and L-arginine, which are responsible for intake by SLC7A5/SLC3A2 heterodimers, can act as signaling molecules to activate mTORC1 and thus mTOR signaling pathway (McCracken and Edinger 2013). Previous studies have observed that mTORC1 is overactive in various types of cancers, such as breast cancer and colon cancer, and confirmed that mTOR pathway plays an obvious role in promoting the development and occurrence of cancer (Park et al. 2014; Saxton and Sabatini 2017). In addition to activating mTORC1, SLC7A5 can also facilitate MYC and EZH2 signaling in cancer cells, while SLC3A2 has also been confirmed to facilitate uncontrolled proliferation of cancer cell through AKT, MAPK, and cell cycle-related P21/P27 signaling pathways (Zhao et al. 2022). Maimaiti et al. found that SLC7A5 protein level was obviously higher in T24 cells than that in normal cells, and SLC7A5 inhibitor JPH203 can inhibit the phosphorylation of MAPK/Erk, AKT, p70S6K, and 4EBP-1 through IGFBP-5 to affect T24 cell proliferation, migration, and invasion (Maimaiti et al. 2020). We compared the multi-omics sequencing data in SV-HUC-1 and MC-SV-HUC T2 cells and found that SLC3A2 and SLC7A5 promoted the malignant phenotype of 3-MC transformed cells by function assays in vivo and in vitro. In addition, our data showed that SLC3A2 and SLC7A5 were not altered in transcriptome level but up-regulated in m^6^A translation and protein levels in MC-SV-HUC T2, a cell malignant transformed by chemical carcinogen 3-MC. We also identified the m^6^A modification sites: the SLC3A2-76 locus and SLC7A5-1134 locus. The current hypothesis suggested that m^6^A modification on these sites might affect the translation efficiency based on our findings. To explore this conjecture, m^6^A modification levels were regulated globally by knocked down or overexpressed methyltransferase or demethylase, and the results showed mRNA levels of SLC3A2 and SLC7A5 were unchanged while the translation levels and protein expression were significantly altered.

**Fig. 6:**
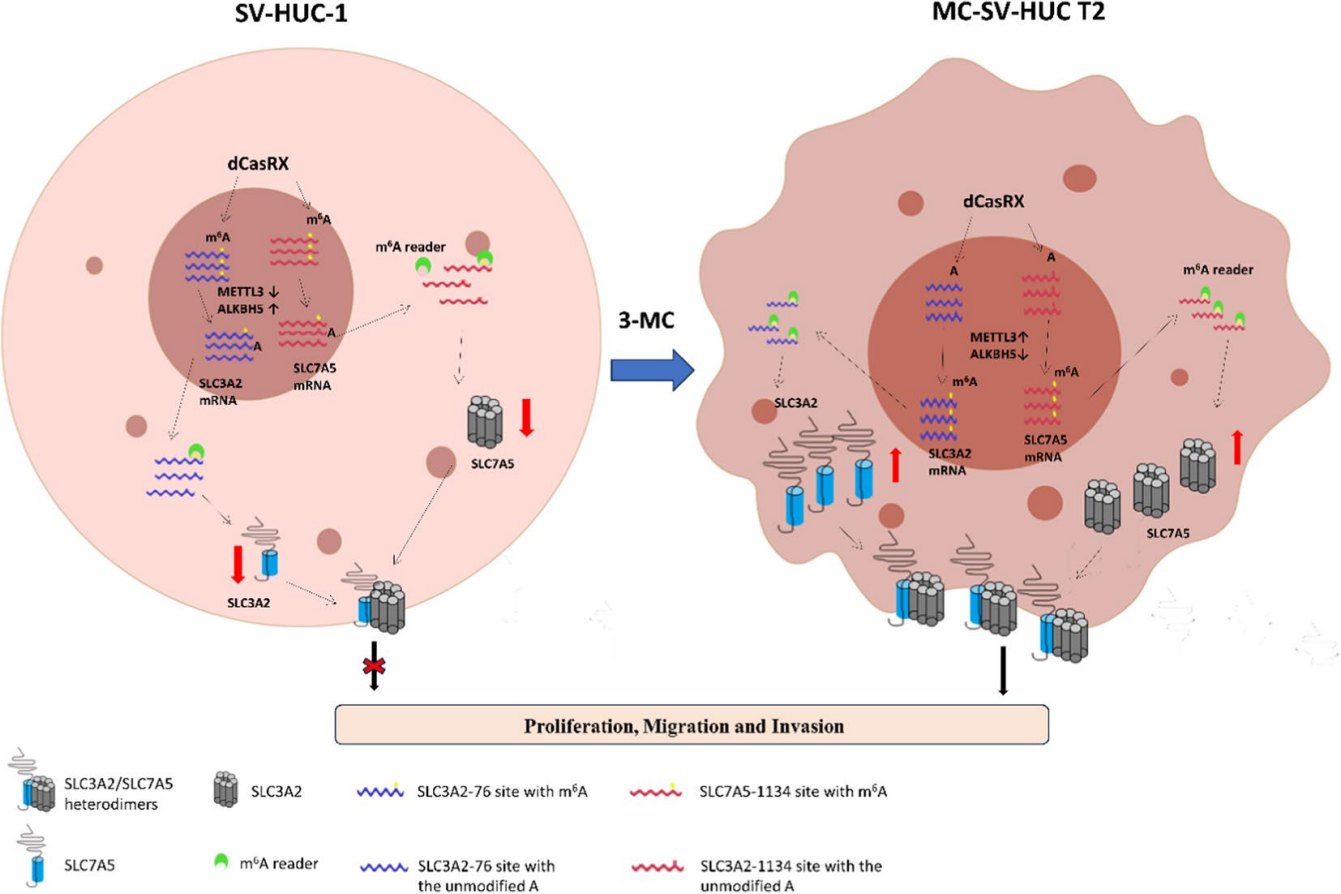
The schematic model of the role and underlying mechanism of m6A-mediated SLC3A2/SLC7A5 translation in 3-MC-induced uroepithelial transformation

Previous studies have found that the methyltransferase METTL3 and demethylase ALKBH5 are aberrantly expressed in bladder cancer cells and are involved in the development of bladder cancer. Cas13d, the shortest known Cas13 protein, is a single crRNA-guided ribonuclease that can target and cleave specific single-stranded RNA targets (Yan et al. 2018). Cas13d can be inserted into vectors (e.g., AAV) for in vivo application and combined with CRISPR arrays encoding numerous gRNAs for multi-point targeting due to its modest size. Cas13d has no substantial flanking sequence dependence and is active in crRNAs that are tiled with the target RNA, implying that its sgRNA design is more adaptable and capable of targeting practically any target RNA (Wilson et al. 2020). This implies that its sgRNA design is more adaptable and can target virtually any single-stranded RNA sequence. CasRx is a variant obtained by fusing Cas13d from the yellow ruminal coccus strain XPD3002 with the nuclear localization sequence NLS (Cox et al. 2017). Compared with RNA interference, CRISPR interference has higher silencing efficiency and greater specificity in eukaryotic cells and remains highly active in adult animals as well as high activity of targeted silencing RNAs in adult animals (He et al. 2020). Previous research has discovered that the process of catalytically inactivated dCasRx targeting mRNA does not interfere with translation, implying that targeting dCasRx to specific coding and non-coding areas in transcripts can be utilized to analyze and edit RNA (Konermann et al. 2018). Furthermore, because CasRx processing of gRNA is not dependent on cleaving the HEPN structural domain of the target RNA, dCasRx, which lacks cleavage enzyme activity, can still be used for multiplexing with CRISPR arrays. We fused m^6^A regulators and dCasRx to obtain dCasRx-METTL3 targeted m^6^A modification and dCasRx-ALKBH5 targeted de-modification systems for targeted regulation of m^6^A modification at given single loci of target mRNA (Ying et al. 2023). In this way, we found that changes in protein expression caused by changes in site-specific m^6^A levels were accompanied by unchanged in mRNA, implying that METTL3 and ALKBH5 mediated m^6^A alterations at the SLC3A2-76 and SLC7A5-1134 loci do increase translation efficiency (Fig. 6).

In summary, this work employed comprehensive multi-omics analysis to screen novel research targets and then used in vitro and in vivo functional assays, gene knockout or overexpression, and m^6^A-targeted alteration to disclose a new mechanism of 3-MC-induced malignant transformation of the urinary epithelium. During the malignant transformation, dysregulation of METTL3/ALKBH5 induced the up-regulation of m^6^A levels on SLC3A2/SLC7A5 mRNA in cells, which promoted translation and expression of SLC3A2/SLC7A5, and thus accelerated bladder cancer growth and metastasis. This suggests that m^6^A modification on mRNA can affect the translation efficiency rather than stability of SLC3A2 and SLC7A5 mRNA. However, the downstream pathways of SLC3A2/SLC7A5 and metabolomics changes during 3-MC-induced malignant transformation of urothelial cells need to be further investigated. Currently, a variety of potential therapeutic agents inhibiting these targets have been developed and investigated for the treatment of tumors. For example, STM2457, a small molecule inhibitor of METTL3 with in vivo activity, was shown to effectively disrupt the proliferation and expansion of leukemia cells, thereby inhibiting cancer progression in various mouse models of acute myeloid leukemia (Yankova et al. 2021). STC-15 (S TORM Therapeutics, NCT05584111) has begun to be tested in the clinic, which is the first RNA epigenetic anti-cancer drug project moving into clinical trials in the world. Moreover, SLC7A5 inhibitors R-OKY-034F (PRN-jRCT2051200105, JPRN-UMIN000036395, JPRN-jRCT2051190003) and Nanvuranlat (JPRN-UMIN000016546, JPRN-UMIN000034080) are also under clinical development or have been completed clinical trials (Nishikubo et al. 2023). Altogether, our data identify METTL3/ALKBH5 and SLC3A2/SLC7A5 as the potential therapeutic targets and pave the way for future development of therapy for bladder cancer related to PAHs.

## Supplementary information

ESM 1 (DOCX 2137 kb)

## Data Availability

The datasets used and/or analyzed during the current study are available from the corresponding author on reasonable request. The RNA-Seq data are deposited at the National Center for Biotechnology Information (NCBI) Sequence Read Archive with the accession PRJNA733588. The Ribo-Seq data are stored into the NCBI Sequence Read Archive with accession PRJNA738263. The proteomics data have been stored in the PRIDE archive with accession PXD026559. MeRIP-seq data are deposited at the Gene Expression Omnibus database with the accession GSE112970.

## Abbreviations

AhR: Aryl hydrocarbon receptor
CAM: Cell adhesion molecules
CC: Cellular component
DEG: Differentially expressed genes
DEP: Differentially expressed proteins
ER: Endoplasmic reticulum
FA: Formic acid
GO: Gene Ontology
MF: Molecular function
PAH: Polycyclic aromatic hydrocarbons
QC: Quality control
