# Supplementary material for: m6A modification mediates SLC3A2/SLC7A5 translation in 3-methylcholanthrene-induced uroepithelial transformation
Source: Cell Biol Toxicol. 2024 Jan 25;40(1):5. doi: 10.1007/s10565-024-09846-9 (PMC10808315; doi:10.1007/s10565-024-09846-9)
Supplement: Supplementary file 1 — (DOCX 2137 kb) [file 10565_2024_9846_MOESM1_ESM.docx]

Supplementary Materials for

**m^6^A modification mediates SLC3A2/SLC7A5 translation in 3-methylcholanthrene -induced uroepithelial transformation**

Bixia Liu^1^, Yifan Lv^2^, Wenyu Hu^1^, Yapeng Huang^1^, Xiaoling Ying^3^, Cong Chen^1^, Haiqing Zhang^1^, Weidong Ji^1, *^

*Corresponding author: Weidong Ji, [jiweidong@mail.sysu.edu.cn](mailto:jiweidong@mail.sysu.edu.cn)

**This file includes:**

Supplementary Methods and materials: 1-10

Supplementary Figures: Fig. S1 to S4

Supplementary Tables: Table S1 to S4

**Supplementary Methods** **and materials**

1. **Cell culture**

Human uroepithelial cells (SV-HUC-1) and 3-MC-transformed SV-HUC-1 cells (MC SV-HUC T2) were obtained from the American Type Culture Collection (Manassas). SV-HUC-1 Cells and MC SV-HUC T2 cells were cultured in Ham's F-12K (Kaighn's) medium (Gibco), and 293T cells were cultured in DMEM (Gibco) medium. The medium was supplemented with 10% fetal bovine serum (Gibco) and 1% Penicillin-Streptomycin (Gibco). All the cells were cultured in a humidified atmosphere comprising 5% CO2 at 37°C. Cells were assessed for Mycoplasma contamination using a PCR-based universal Mycoplasma detection kit (Abcam).

1. **Transcriptome analysis**

Total RNA was extracted from the cells using TRIzol reagent (Invitrogen Inc), and the genomic DNA was removed by DNase I. The mRNA was enriched by Oligo(dT) magnetic beads and fragmented at the right temperature in a thermomixer. The cDNA library was generated using Illumina TruSeq RNA Sample Preparation Kit (Illumina) and sequenced on an Illumina HiSeq 2000 platform (Illumina).

After checking for quality using the FastQC program (version: 0.11.3) (<http://www.bioinf-ormatics>.babraham.ac.uk /projects/fastqc/), unqualified reads (such as low-quality reads and unpaired-end reads) were removed to clean the raw reads generated from sequencing. The clean reads were mapped to the human genome using the HISAT2 (version 2.0.5)(Kim et al., 2015). After passing the second quality control (QC of alignment), reads per kilobase million mapped reads were calculated to compare the expression level of genes between two groups. DEGs with FDR ≤0.001 and log2 fold change >1 or < -1 among groups were screened out using R (version 4.0.0) (The R Development Core Team, Vienna, Austria). Finally, GO and KEGG pathway significant enrichment analysis were performed based on the DEGs using the online tool DAVID (https://david.ncifcrf.gov/). GO terms and KEGG pathways with a Bonferroni corrected *P* value of <0.05 were considered significantly enriched function annotations.

1. **Proteomic analysis**

Protein was extracted from the cells and protein concentrations assessed using the Pierce BCA Protein Assay Kit (Thermo Fisher). The proteins were enzymolized as described previously (Wisniewski et al., 2009). Enzymatic peptides were labeled with iTRAQ using the iTRAQ 8 plex kit (AB Sciex). All labeled peptides were then mixed, centrifuged, concentrated, and dried.

A 110-μl mobile phase A mixture (contents: 10 mM ammonium formate, 5% acetonitrile aqueous solution, pH 10.0) was used to solubilize the dried samples. The samples were fractionated using a high-performance liquid chromatography system with high pH reversed-phase chromatography (Agilent Technology). The samples produced by chromatography were frozen, dried, and resuspended in 0.1% formic acid (FA). They were subjected to online nanoliter scale liquid chromatography-tandem mass spectrometry analysis. In brief, the samples were loaded into a 3 μm, 120 Å C18 trap column of a two-dimensional nanoLC-Ultra^TM^ system (75 μm × 15 cm; AB Sciex). Sample pumps were desalted with a phase A mixture (2% acetonitrile, 0.1% FA, v/v) at a flow rate of 3.0 µl/min for 10 min. The peptides were then separated using a 70 min gradient from 5% to 40% phase B (98% acetonitrile, 0.1% FA, v/v). Meanwhile, the eluents were scanned in the information dependent acquisition mode using the TripleTOF 5600 plus system (AB Sciex).

The tandem mass spectra of samples were searched against the HUMAN non-redundant protein database (Uniprot_HUMAN_2017_04), which contains 20,191 protein sequences, using the Sciex ProteinPilot software (version 4.5). The parent mass tolerance was set to 20 ppm and the precursor mass tolerance was set to 0.1 Da. In addition, protein identification was performed using a thorough analysis mode with a peptide spectrum matching false discovery rate set to 1%, iTRAQ 8-plex labeled quantification, and trypsin as the enzyme for specific digestion. Only proteins with fold changes >1.5 and *P*-values <0.05 were considered as DEPs. Function annotation and pathway analysis of proteins were performed through GO analysis and KEGG analysis. The interactive relationship of the proteins was retrieved from the STRING database (http://string-db.org/cgi/).

1. **m^6^A profiling**

Total RNA was extracted from the cells using TRIzol reagent and enriched for mRNA using Oligo(dT) magnetic beads following the instructions for the Dynabeads^®^ mRNA Purification Kit (sigma MRN10). The mRNA was then chemically fragmented using an RNA fragmentation reagent (Invitrogen) and purified using the Oligo Clean & Concentrator^TM^ Zymo D4061 kit (ZYMO Research). Approximately more than 200 ng of fragmented mRNA was obtained, 100 ng of which was subjected to m^6^A immunoprecipitation (IP). In brief, the fragmented mRNA was subjected to incubation with anti-m^6^A antibody (no. 202003; Synaptic Systems) in IP buffer (50 mM Tris-HCl, pH 7.4, 750 mM NaCl, and 0.5% Igepal CA-630) supplemented with bovine serum albumin (0.5 μg/μl) for 3 h at 4°C. The mixture was incubated with Dynabeads protein A beads at 4°C by rotating for another 3 h. The bound RNA fragments were eluted from the beads using an elution buffer (1× IP buffer and 6.7 mM m^6^A). RNA was isolated from the eluate by ethanol precipitation. Sequencing was performed on an Illumina HiSeq^TM^ 2000/ MiSeq^TM^ platform (Illumina).

The resulting image data files were converted to sequenced reads via CASAVA base calling analysis. The raw reads were cleaned by removing reads that contained adapter contamination, low-quality bases (Q ≤5), and had a high ratio N (≥10%). The clean reads were mapped to the human reference genome using TopHat. Mapped reads of IP and input libraries were scanned using the R package *exomePeak*. The R package *edgeR* was used to analyze the differential expression of genes, including GO functional analysis and KEGG pathway analysis.

1. **Ribosome profiling sequencing**

The harvested cells were washed, collected and then lysed with lysis buffer. After complete lysis, the supernatant was collected by centrifugation at 4°C. The supernatant was loaded onto a sucrose gradient column and the pellet containing the ribosomes was separated by ultracentrifugating (36 000 rpm; 2.5 h; 4°C). Then the RNA fragments without ribosome protection were digested with a purified ribonuclease (Escherichia coli RNase I). The ribosome-protected RNA fragments (RPF) of about 30 bp were converted to DNA and analyzed by next-generation sequencing technology on the Illumina GAII system according to the manufacturer’s protocol. The row reads were abtained from deep sequencing and cleaned by discarding low-quality reads. Bowtie2(2.2.8) short-read alignment program was used to align clean reads to rRNA reference and genomic reference for discarding rRNA, tRNA, snoRNA, snRNA and miRNA. Comparative analysis and gene statistics were performed on the unaligned reads. The RSEM software calculated the number of reads at the Ribo-seq level in the ORF region of the coding gene and converts it to the FPKM value, so as to obtain the expression of the gene at the translation level. Differential gene expression in ribosome profiling data were analyzed by edgeR.

1. **Cell proliferation assay**

5000 cells were seeded per well in 96-well plates and cultured in humidified atmosphere comprising 5% CO_2_ at 37°C. At equal intervals (24 h, 48 h, 72 h, 96 h and 120 h), 20 μl CellTiter 96® AQueous One Solution Reagent (Promega) was added to each well. After incubation for 2 hours, the absorbance of wells was dectected at 490 nm using the SYNERGY H1 microplate reader (Bio Tek).

1. **Wound healing assay**

1$\times$10^6^ cells were seeded per well in 48-well culture plate and cultured in humidified atmosphere comprising 5% CO_2_ at 37°C. After examining the confluency of cells as a monolayer by a light microscopy, wounds were made in the center of the plates. In order to limit cell growth and encourage migration, the medium was changed to one containing 1% fetal bovine serum (FBS). The cells were imaged directly after making the wound and every 2 h for a total duration of 24 h.

1. **Cell invasion assay**

Mix 8~12 mg/ml Matrigel matrix (Corning) and serum-free medium at a ratio of 1:8~10 on ice, and carefully add 50 μl of diluted Matrigel to each Transwell chamber in 24-well culture plate. Then the chamber was incubated in 37°C until the gel solidified. 1×10^5^ cells in 200 μl serum-free cell suspension was added to the upper part of the Transwell chamber, and 600 μl 20% FBS culture medium was added to the lower part. The cells were cultured for 24 hours at 37°C. The invaded cells were fixed with 100% methanol for 15-30 minutes and stained with 0.1% crystal violet solution for 30 min at room temperature. After washing with PBS and wiping the cells on the surface of the chamber with a cotton swab, the invaded cells were counted under a ZEISS Axio Imager. Z2 microscope. 5 fields of view under a high-power lens (200×) were randomly selected to count, and the average value was taken.

1. **Western blot**

Protein was extracted from SV-HUC-1 and MC SV-HUC T2 cells. 35 μg of protein was loaded and separated by 10% sodium dodecyl sulfate-polyacrylamide gel electrophoresis, and then transferred electronically to polyvinylidene difluoride membranes (Bio-Rad). The membranes were blocked with 5% BSA for 1 hour and incubated with the 1:1,000 diluted specific antibody [anti-CD98 antibody (ab307587, Abcam), anti-LAT1 antibody (ab305251, Abcam), anti-GAPDH (#2118L, Cell Signaling Technolog), or anti-β-actin (#4970L, Cell Signaling Technolog)] at 4 ℃ overnight. And then they were incubated with the 1:5,000 diluted anti-rabbit secondary antibody (#7074S, Cell Signaling Technology). At last, membranes were scanned with an imaging system (ChemiDOC™ MP, Bio-Rad).

1. **RT-qPCR**

Total RNA was synthesized into cDNA using HiScript II Q RT SuperMix for qPCR (Vazyme Biotech) in conformity with the manufacturer's instructions. Then cDNA was diluted and mixed with specific reverse primers (see Supplementary Table 1) and Fast SYBR Green PCR Master Mix (Thermo Fisher Scientific) according to the manufacturer’s instructions. The mixture performed quantitative real-time PCR (qPCR) on Step-One Fast Real-time PCR System (Applied Biosystems, Carlsbad, CA, USA). At last, obtained post-run cycle threshold (CT) values were converted to Fold Change (FC) using the 2^^-△△CT^ method, normalized to β-actin.

**Supplementary Figures**


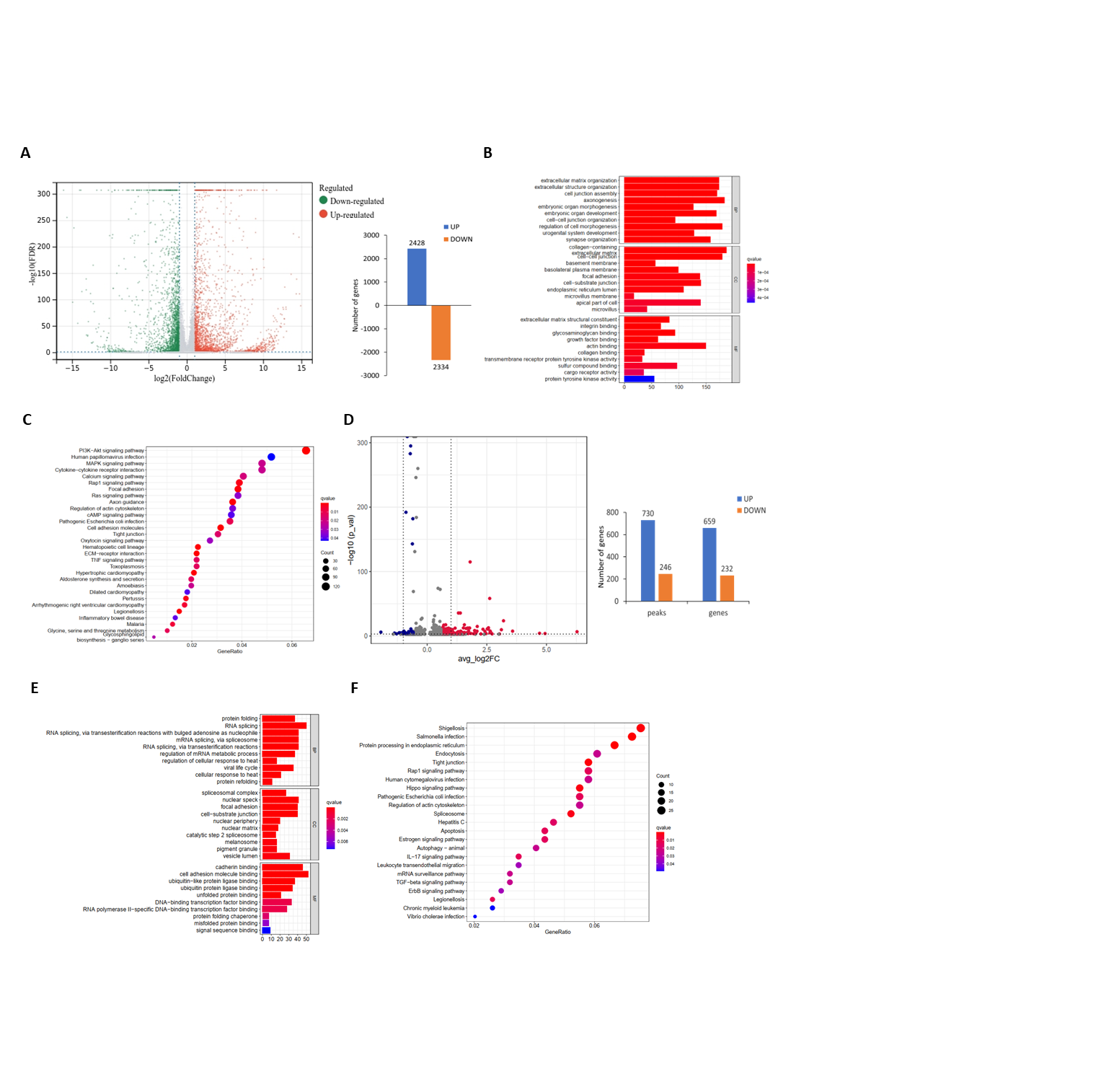


**Fig. S1. Analysis of the DEGs and significantly different m^6^A peaks between MC SV-HUC T2 and SV-HUC-1**

**A.** Volcano plot and histogram of differences in mRNA level between MC SV-HUC T2 and SV-HUC-1. Y-axis: the log10 expression of MC SV-HUC T2; X-axis: the log10 expression of SV-HUC-1. Each dot in the graph represents a gene. Red and green dots represent significantly expressed genes and indicate that the gene expression is upregulated and downregulated, respectively, in MC SV-HUC T2 compared to SV-HUC-1. The blue dots indicate that there are no significantly different genes between MC SV-HUC T2 and SV-HUC-1. **B.** GO function analysis of the DEGs between MC SV-HUC T2 and SV-HUC-1. Y-axis: the GO term enriched in three ontologies; X-axis: the number of genes enriched on the GO term. **C.** KEGG enrichment analysis of the DEGs between MC SV-HUC T2 and SV-HUC-1. Y-axis: name of the signaling pathway; X-axis: percentage of the number of DEGs assigned to a term among the total number of genes annotated in the pathway; Bubble size: number of DEGs assigned to a pathway; Color: enriched adjusted P value; Different colors represent different significance levels. **D.** Volcano plot and histogram of differences in m^6^A level between MC SV-HUC T2 and SV-HUC-1. **E.** Major enrichment and meaningful GO terms of m^6^A peaks. **F.** Significant pathways (*P* <0.05) are shown.

**
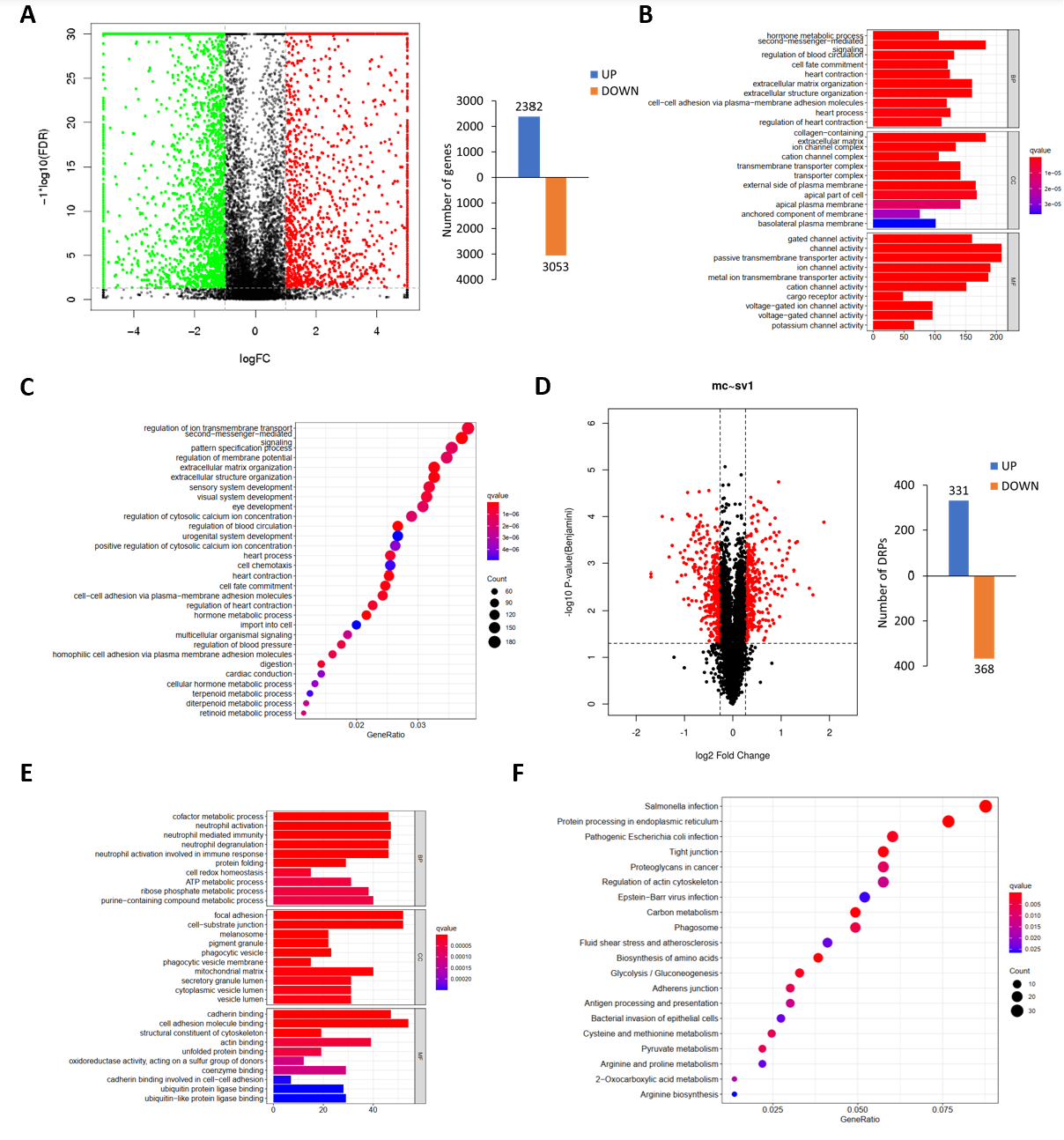
**

**Fig. S2. Analysis of up- and downregulated DTGs and DEPs in MC SV-HUC T2 compared to SV-HUC-1**

1. Volcano plot and histogram of differences in translation level between MC SV-HUC T2 and SV-HUC-1.
2. GO terms of DTGs between MC SV-HUC T2 and SV-HUC-1 were highly enriched.
3. KEGG pathway enrichment analysis based on the DTGs of MC SV-HUC T2 and SV-HUC-1.
4. Volcano plots with differentially regulated protein groups marked in red. And histogram of differences in protein level between MC SV-HUC T2 and SV-HUC-1.
5. GO terms of DEPs between MC SV-HUC T2 and SV-HUC-1 were highly enriched.
6. KEGG pathway enrichment analysis based on the DEPs of MC SV-HUC T2 and SV-HUC-1.


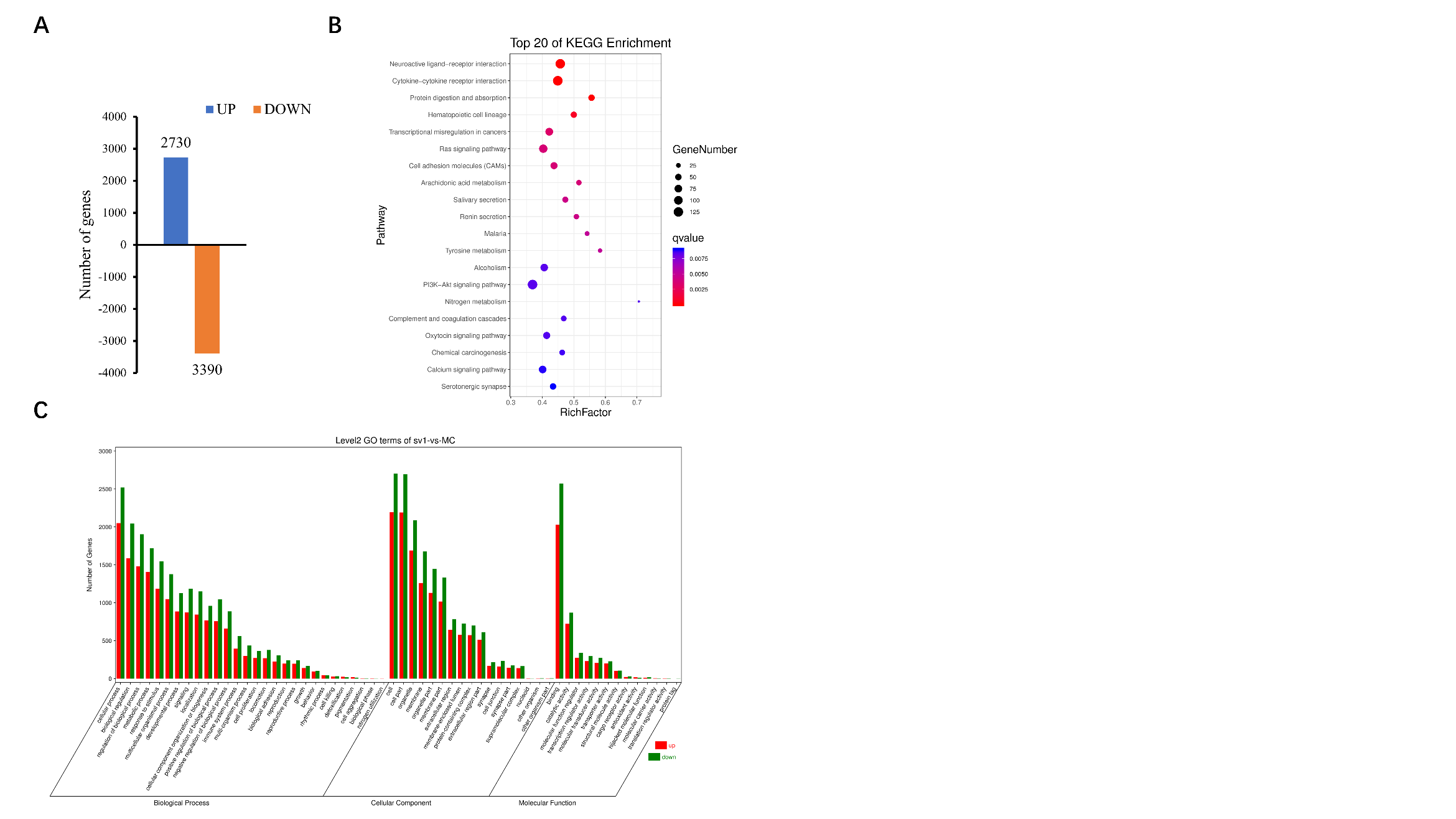


**Fig. S3. Analysis of up- and downregulated DTEGs in MC SV-HUC T2 compared to SV-HUC-1**

1. Histogram of differences in TE between MC SV-HUC T2 and SV-HUC-1.
2. KEGG pathway enrichment analysis based on the DTGs of MC SV-HUC T2 and SV-HUC-1.
3. GO terms of DTEGs between MC SV-HUC T2 and SV-HUC-1 were highly enriched.


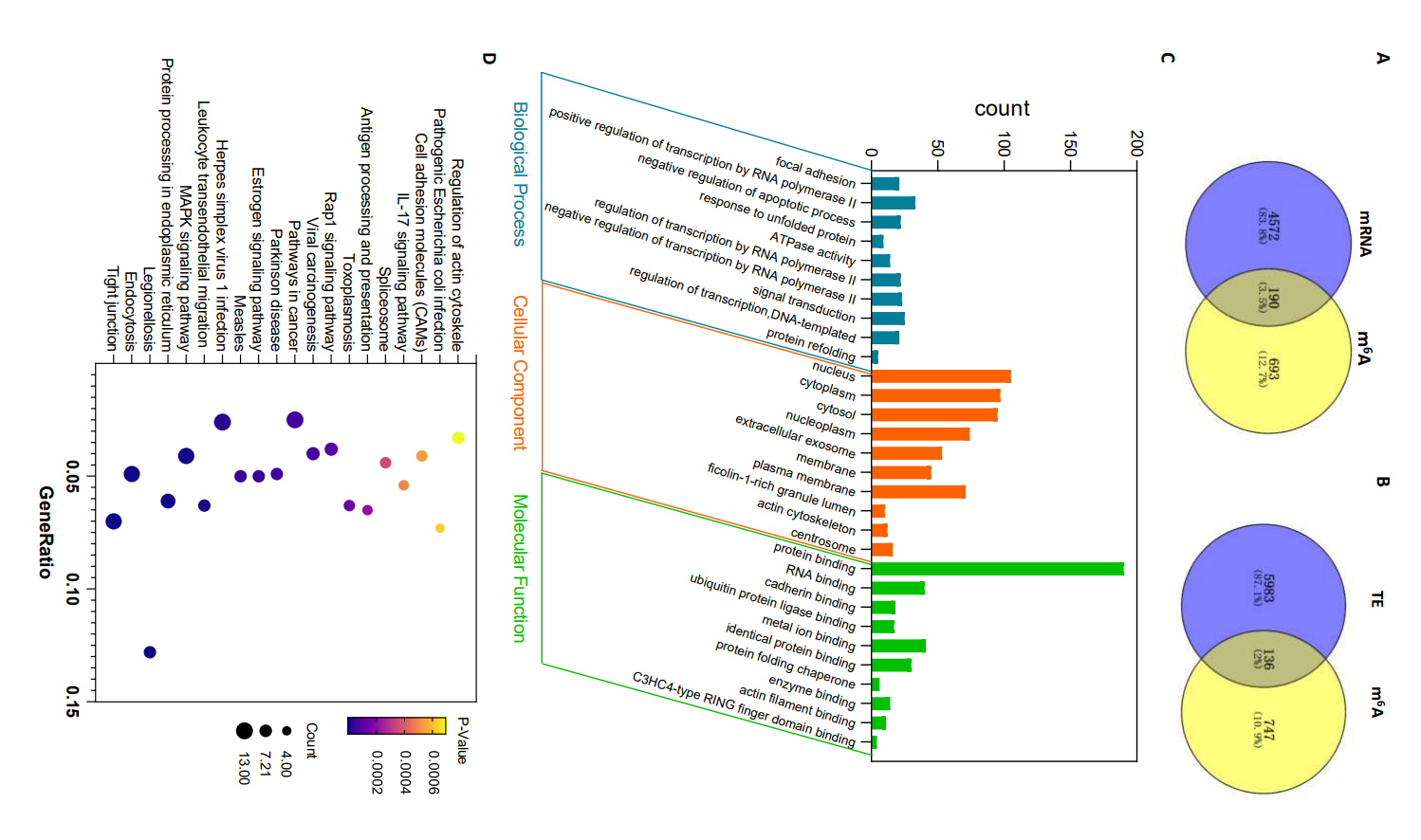


**Fig. S4. Integrated analysis of differences at mRNA or TE and m^6^A levels.**

1. Venn diagram of differences in mRNA and m^6^A levels.
2. Venn diagram of differences in TE and m^6^A levels.
3. GO terms of the co-changed genes at mRNA or TE and m^6^A levels were highly enriched.
4. KEGG pathway enrichment analysis based on the co-changed genes at mRNA or TE and m^6^A levels.

**Supplementary Tables**

**Table S1. PCR primer sequence of SLC3A2 and SLC7A5.**

| Name | Seqences（5' to 3'） |
| --- | --- |
| SLC3A2-Forward primer | CGG GATCCATGGAGCTACAGCCTCCTGA |
| SLC3A2-Reverse primer | ATAAGAATGCGGCCGCTCAGGCCGCGTAGGGGA |
| SLC7A5-Forward primer | CGGGATCCATGGCGGGTGCGGGCCCGAAGC |
| SLC7A5-Reverse primer | ATAAGAATGCGGCCGCCTATGTCTCCTGGGGGACCACC |

**Table S2. gRNAs sequence of SLC3A2 and SLC7A5 for constructing knockout plasmid.**

| Name | Seqences（5' to 3'） |
| --- | --- |
| SLC3A2-gRNA oligo-1 | aaacTGTTTTTAAAAAAGAGAAAAATCAGAAGCC |
| SLC3A2-gRNA oligo-2 | aaaaGGCTTCTGATTTTTCTCTTTTTTAAAAACA |
| SLC7A5-gRNA oligo-1 | aaacTGCTGGTGGTCCTCGGCCTCCAGACCGTGG |
| SLC7A5-gRNA oligo-2 | aaaaCCACGGTCTGGAGGCCGAGGACCACCAGCA |

**Table S3. gRNAs sequence of SLC3A2 and SLC7A5 for programmable m^6^A modification.**

| Name | Seqences（5' to 3'） |
| --- | --- |
| SLC3A2-gRNA oligo-1 | aaacTGTTTTTAAAAAAGAGAAAAATCAGAAGCC |
| SLC3A2-gRNA oligo-2 | aaaaGGCTTCTGATTTTTCTCTTTTTTAAAAACA |
| SLC7A5-gRNA oligo-1 | aaacTGCTGGTGGTCCTCGGCCTCCAGACCGTGG |
| SLC7A5-gRNA oligo-2 | aaaaCCACGGTCTGGAGGCCGAGGACCACCAGCA |

**Table S4. Probe L and Probe R of SLC3A2 and SLC7A5 for single-base mapping of m^6^A sites.**

| Name | Seqences（5' to 3'） |
| --- | --- |
| SLC3A2-76 Probe L | AGGCCCTTTGGCTTCTGATT |
| SLC3A2-76 Probe R | CCCTATTTGGGGGTTCACTCA |
| SLC7A5-842 Probe L | CCTCGGGATGCCGTTCTG |
| SLC7A5-842 Probe R | CGGCTCCCTGTATCCTTGAG |
| SLC7A5-1134 Probe L | GAATTCCACTCGTCCAAGGG |
| SLC7A5-1134 Probe R | CACCAGCTGCTGTCCTTATT |
| SLC7A5-1246 Probe L | TTTATTCACCCCTGCCCCGTT |
| SLC7A5-1246 Probe R | ATGCAGGTCCTATGAGCAGAC |
